# Cervicovaginal *Gardnerella* sialidase-encoding gene in persistent human papillomavirus infection

**DOI:** 10.1038/s41598-023-41469-8

**Published:** 2023-08-31

**Authors:** Juliano Novak, Rafael Belleti, Gabriel Vitor da Silva Pinto, Aline do Nascimento Bolpetti, Márcia Guimarães da Silva, Camila Marconi

**Affiliations:** 1 Department of Pathology, Botucatu Medical School, UNESP, São Paulo State University, São Paulo, Brazil; 2 Department of Basic Pathology, Sector of Biologic Science, UFPR, Universidade Federal do Paraná, Curitiba, Brazil

**Keywords:** Cancer, Microbiology, Molecular biology, Diseases, Risk factors

## Abstract

Disturbed vaginal microbiota have a role in the persistence of high-oncogenic-risk human papillomavirus (hrHPV) and *Gardnerella* spp. is closely related with this condition. Such bacteria are the major source of cervicovaginal sialidases, important for microbiota alterations. The sialidase-encoding gene nanH3 is account for their sialidase activity. Thus, a subset of 212 women positive for hrHPV at the first visit were included in the analysis of the current study aiming to compare the loads of nanH3 in cervicovaginal fluid (CFV) of women with persistent hrHPV infection and with those cleared the infection after a year. Participants were assigned to two study groups named “persistence” (n = 124, 53.22%) or “clearance” (n = 88, 37.77%), according to the HPV status upon enrollment and follow-up. Absolute quantification of nanH3 gene was performed using quantitative real-time PCR (qPCR). Persistence and clearance group did not show statistical difference in the load of nanH3 gene (p = 0.19). When considering the subset of women with HPV16, differences in number of copies of nanh3 gene was observed between the persistent (7.39E+08 copies/μL) and clearance group (2.85E+07 copies/μL) (p = 0.007). Therefore, baseline loads of nanH3 gene is increased in women that persist with cervical HPV16 infection after 12 months.

## Introduction

The cervical infection by human papillomavirus (HPV) is the most frequent sexually transmitted infection (STI) worldwide^1,2^ and the persistence of cervical infections by high-risk-HPV (hrHPV) for long periods of time cause virtually all precursor lesions and cervical cancers^3^. Despite that, the majority of the cases of cervical HPV infection are cleared within 2 years^4,5^, being the immune responses important for clearance^6^.

Factors associated with developing cervical lesion and cancer include smoking^7^, hormonal contraceptive use^8,9^, and parity^10^. In addition, the local cervical microenvironment, including the vaginal microbiota, may also influence the natural history of HPV infection^11^. Therefore, a *Lactobacillus*-deprived vaginal microbiota, as in bacterial vaginosis (BV), have been associated with persistent HPV infection and lesion progression^12–14^.

Bacterial vaginosis is a polymicrobial dysbiosis, which occurs by the replacement of beneficial *Lactobacillus* and increased anaerobic and facultative anaerobic bacteria, of which, *Gardnerella* spp. that is present in nearly all cases^15,16^. Sialidase production is one of the most important virulence factors of *Gardnerella* spp.^17,18^. Among the deleterious effects of bacterial sialidases, it is the degradation of several protective factors of the vaginal mucosa and contribution to the exfoliation and detachment of vaginal epithelial cells^19^ facilitating bacterial adhesion to the epithelium and biofilm formation^20–22^, a condition already associated with persistence of BV^23^.

At first, the putative sialidase gene, nanH1 (sialidase A gene), was identified in *Gardnerella* spp. and was thought to be the gene responsible for sialidase production^24^. However, the recent studies concluded that nanH2 and nanH3 account for the sialidase activity observed in cultured *G. vaginalis*^25^. Besides that, nanH2 is less prevalent in *Gardnerella* isolates and always detected when in the presence of nanH3^18,25^.

Until very recently, *Gardnerella* was one single specie genus but now three other species in addition to *G. vaginalis* (*G. leopoldii*, *G. piotii* and *G. swidsinskii*) were described^26^. Interestingly, sialidase genes nanH2 and nanH3 were only detected in *G. piotii* and in a subset of *G.vaginalis* isolates^18^.

Considering the importance of better understanding the relationship between the bacterial components of the vaginal microbiota and the outcome of hrHPV infection, the aim of this study was to compare the loads of the sialidase-encoding gene nanH3 of *Gardnerella* spp. in the cervicovaginal fluid of women between persistent hrHPV infection and those who cleared the infection after a 12-month period.

## Results

Table 1 displays the sociodemographic, behavioral and clinical characteristics of the participants, according to the study groups. The groups showed differences in few variables such as age (p = 0.04), and the number of sex (p < 0.0001). When considering the number of hrHPV genotypes detected, persistent group had more mixed infection than clearance group (p = 0.002). In relation to the HPV genotypes, HPV52 differed between the groups when considering single or mixed infection (p = 0.01).

**Table 1:** Sociodemographic, behavioral and clinical characteristics of the participants, according to the study groups.

Variable	Outcome of hrHPV infection		
	Clearance (88)	Persistence (124)	P value
Age^#^	29 (17–50)	26.5 (17–50)	**0.04**
Ethnicity*			0.52
Black	35 (39.77)	59 (47.58)	
White	51 (57.95)	62 (50.00)	
Other	2 (2.27)	3 (2.42)	
Living with partner^$^			0.89
No	41 (46.59)	60 (48.39)	
Yes	47 (53.41)	64 (51.61)	
Years at school^#^	11 (1–16)	11 (3–17)	0.14
Smoking^$^			0.66
No	57 (64.77)	85 (68.55)	
Yes	31 (35.23)	39 (31.45)	
Douching^$^			1.00
No	84 (95.45)	117 (94.35)	
Yes	4 (4.55)	7 (5.65)	
Hormonal contraceptive use^$^			0.14
No	33 (37.50)	34 (27.42)	
Yes	55 (62.50)	90 (72.58)	
Condom use^$^			0.88
No	63 (71.59)	87 (70.16)	
Yes	25 (28.41)	37 (29.84)	
Sex partners^#^	0 (1–15)	3 (1–100)	** < 0.0001**
Sex partners (last year)^#^	1 (0–2)	1 (0–12)	0.26
Vaginal abnormal discharge^#^			0.25
No	27 (30.68)	48 (38.71)	
Yes	61 (69.32)	76 (61.29)	
Nugent*			0.59
Normal	50 (56.82)	68 (54.84)	
Intermediate	10 (11.36)	10 (8.06)	
BV	28 (31.82)	46 (37.10)	
Number of hrHPV genotypes^#^	1 (1–3)	1 (1–5)	**0.002**
HPV16^$^			1.00
Single	12 (13.64)	31 (25.00)	
Mixed	5 (5.68)	16 (12.90)	
HPV31^$^			0.43
Single	6 (6.82)	9 (7.26)	
Mixed	3 (6.41)	10 (8.06)	
HPV51^$^			0.12
Single	12 (13.64)	5 (4.03)	
Mixed	1 (1.14)	4 (3.23)	
HPV52^$^			**0.01**
Single	9 (10.23)	6 (4.84)	
Mixed	1 (1.14)	10 (8.06)	
HPV58^$^			1.00
Single	4 (4.55)	7 (5.65)	
Mixed	3 (3.41)	8 (6.45)	

For all analysis P-value < 0.05 was considered as significant.

*Chi-square test.

^#^Mann–Whitney test, expressed by median (minimum–maximum).

^$^Fisher’s exact test.

Significant values are in bold.

The most frequently genotype detected in the population (showed in Supplementary Table S1), was HPV16, followed by HPV31, HPV52, HPV51, HPV58 and HPV45, respectively. Of 233 women included in this study, 21 cleared their HPV genotype detected at baseline and a new genotype was detected at follow-up, therefore these women were not included in the analysis. Thus, clearance group included women without detection of HPV at follow-up (n = 88) and persistence group includes those women showing at least one genotype detected at baseline and follow-up (n = 124). Thus, clearance and persistence rates were 41.50% (n = 88) and 58.49% (n = 124), respectively. For HPV16, rate of clearance was 26.56% (n = 17) and persistence was 73.44% (n = 47).

Regarding the presence of nanH3 gene and the outcome of cervical hrHPV infection displayed on the Table 2, the groups did not show difference in relation to the positivity to the gene. When compared the absolute quantification of nanH3 gene, showed by median (minimum–maximum), in the presence of all hrHPV, no difference in the load was observed between the clearance (1.45E+08 copies/µL; 2.30E+04–6.15E+13) and persistence (9.16E+08 copies/µL; 3.02E+05–1.72E+13) groups (p = 0.19). For five most prevalent genotypes (HPV16, 31, 52, 51 and 58) clearance group showed 1.02E+08 copies/µL (2.30E+04–6.15E+13) and persistence 1.82E+09 copies/µL (3.67E+05–1.72E+13) (p = 0.02). When excluding HPV16, the 5 most prevalent genotypes (HPV31, 52, 51, 58 and 45) there was no difference, the clearance group showed 3.60E+08 copies/µL (2.30E+04–6.15E+13) and persistence 1.36E+09 (3.67E+05–1.72E+13) (p = 0.42). Loads of nanH3 gene differed between the clearance (2.85E+07 ng/µL; 1.90E+05–4.23E+07) and persistence group (7.39E+08 copies/µL; 4.15E+06–4.09E+10) in the presence of HPV16 (p = 0.007) (Table 3).

**Table 2:** Detection of nanH3 gene in the cervicovaginal fluid according to the study groups.

	Detectable nanH3 gene		P value*
	Yes	No	
All hrHPV			0.77
Clearance	32 (36.36)	56 (63.64)	
Persistence	48 (38.71)	76 (61.29)	
5 most prevalent hrHPV genotypes^&^			0.60
Clearance	20 (37.74)	33 (62.26)	
Persistence	38 (42.70)	51 (57.30)	
5 most prevalent hrHPV genotypes, excluding HPV16^$^			0.70
Clearance	19 (38.78)	30 (61.22)	
Persistence	26 (44.07)	33 (55.93)	
HPV16			0.56
Clearance	5 (29.41)	12 (70.59)	
Persistence	19 (40.43)	28 (59.57)	

For all analysis P < 0.05 was considered as significant.

*Fisher’s exact test.

^&^Including HPV16, HPV31, HPV52, HPV51 and HPV58.

^$^Including HPV31, HPV52, HPV51, HPV58 and HPV45.

**Table 3:** Load of nanH3 gene in the cervicovaginal fluid according to the study groups.

	NanH3 loads (copies/µL)^#^		
	Clearance	Persistence	P value
All hrHPV	1.45E+08 (2.30E+04–6.15E+13)	9.16E+08 (3.67E+05–1.72E+13)	0.19
5 most prevalent hrHPV genotypes^&^	1.02E+08 (2.30E+04–4.34E+13)	1.82E+09 (3.67E+05–1.72E+13)	**0.02**
5 most prevalent hrHPV genotypes, excluding HPV16^$^	3.60E+08 (2.30E+04–6.15E+13)	1.36E+09 (3.67E+05–1.72E+13)	0.42
HPV16	2.85E+07 (1.90E+05–4.23E+07)	7.39E+08 (4.15E+06–4.09E+10)	**0.007**

For all analysis P < 0.05 was considered as significant.

^&^Including HPV16, HPV31, HPV52, HPV51 and HPV58.

^$^Including HPV31, HPV52, HPV51, HPV58 and HPV45.

^#^Mann–Whitney Test, expressed by median (minimum–maximum).

Significant values are in bold.

**Figure 1:**
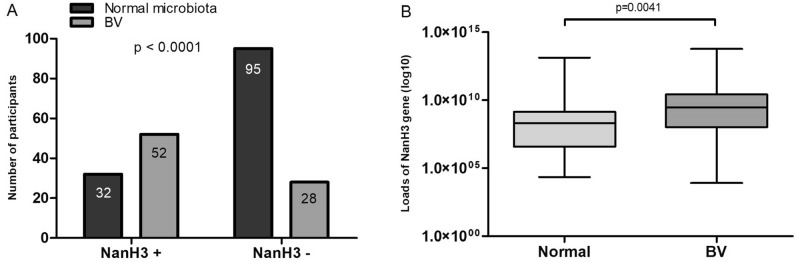
Characteristics of association between nanH3 gene and vaginal microbiota. (**A**) A bar graph illustrating the relationship of nanH3 gene status across the two Nugent score categories (p < 0.0001); (**B**) loads of nanh3 gene compared between normal microbiota and BV (p = 0.0041).

Analysis to test the association between vaginal microbiota and nanH3 gene were also performed. Of the 233 women, 127 (54.51%) had normal microbiota, 21 (9.01%) intermediate and 80 (34.33%) had BV, detected by Nugent scoring. Data of Nugent evaluation were not available for five participants. Among BV positive women, 52/80 (65%) were nanH3 positive, while 28/80 (35%) were nanH3 negative. Thus, sensitivity and specificity of the test was 65% (95% confidence interval, 54–75%) and 75% (66–82%), respectively. Figure 1A displays a bar graph illustrating the relationship of nanH3 gene status across normal and BV Nugent scoring, showing a significant difference between the categories (p < 0.0001). In relation to the loads of nanH3 gene expressed by median (minimum–maximum) in the cervicovaginal fluid shown in Fig. 1B, there was difference between normal microbiota (2.06E+08 copies/µL; 2.30E+04–1.37E+13) and BV (2.91E+09 copies/µL; 8.17E+03–6.15E+13) (p = 0.0041). Due the low representativeness, intermediate microbiota was not considered in this analysis.

## Discussion

Recently our study group showed that women with persistent HPV16 and HPV18 infection have increased baseline loads of *G. vaginalis*^27^. Thus, it was hypothesized that gene encoding sialidase, frequently found in *Gardnerella* spp.^18^ could have a role in the outcome of HPV infection, being a useful marker for hrHPV persistence. Surprisingly, loads of nanH3 gene differed between clearance and persistence group in the presence of 5 most prevalent genotypes, however such difference remains only in the presence of HPV16.

This study showed high rates of HPV persistence, 58.49% for all hrHPV infection and 73.44% when considered only the HPV16. Such rates were similar to the rates showed in another study conducted in Brazil, with 61.8% of persistence rate^28^. Persistent rates in a large retrospective cohort study in China was 42.7% within 24 months^29^, while a multicentric study showed 54.1% of persistence rate over a year in American, Canadian and Brazilian women^30^. In relation to HPV16, the persistence rate was 52.1% after 12 months in Dutch cohort of young women^31^.

In relation to sociodemographic and behavioral characteristics of the study population, few variables differed between the groups. The median of age was higher in clearance group, agreeing with a study that showed that the highest HR-HPV persistence occurred in the 22–27 years old group, whereas clearance increased in women aged 28–33 years^32^. However, other Brazilian study did not show differences of age when evaluating factors associated with clearance and persistence after 24 months^28^. The number of sex partners and the number of detected genotypes differed between the clearance and persistence group. Furthermore, one of five most prevalent HPV genotypes differed among the groups when considering their detection in single or mixed infection. In fact, a previous study showed that HPV genotypes differ in terms of the period for clearance, as women with mixed infections had a longer time to clear their infections compared to those with a single infection^30^. The increasing in number of sex partners could contribute to persistence since it increases the risk of acquiring new HPV genotypes.

Studies on vaginal microbiota have demonstrated the association between *Gardnerella* spp. and persistent hrHPV infection, as well as progression of the cervical lesion^33,34^. Of the results of association between *Gardnerella* spp. with persistence and progression of hrHPV, Usik et al. proposed that in addition to role of *Gardnerella* spp. in the viral persistence, it also contributes for the disruption on vaginal microbiota (i. e. increased bacterial diversity), which also leads to the persistence and progression of HPV infection^34^.

As recently demonstrated, not all newly described species of *Gardnerella* produce sialidase^18^. Therefore, studying the presence of sialidase encoding gene of *Gardnerella* is important for paving the way for further studies focusing in the role of each individual *Gardnerella* sp. for HPV infection and persistence.

To the best of our knowledge, this is the first study to assess the relationship between hrHPV persistence infection and loads of nanH3 gene. It is known that sialidases hydrolyze the sialic acid of the host’s epithelial cells, provides a carbon source to *Gardnerella* allowing their uptake and catabolism and expose binding sites, facilitating bacterial adhesion to the epithelium and further biofilms formation^20–22,35^. Sialidases can still cleave mucin oligosaccharides, that decreased the cervicovaginal mucus viscosity, compromising the physical and biochemical barrier against pathogens^36^. Besides that, sialidases compromise the immune barrier by hydrolyzing immunoglobulin A^35,37^. In the present study, results on the quantification of nanH3 did not differ when included all hrHPV infection grouped, however loads of nanH3 were higher in the cervicovaginal fluid of women with HPV16 persistent.

Studies have been reporting the association of altered vaginal microbiota with HPV infection^12–14,33,34,38^, despite the mechanisms by it can influence in HPV infection remain unknow. It is known that *Lactobacillus* spp. produce several microbicidal factors, including lactic acid^39^, an important agent to acidify the vaginal environment and to control overgrowth of bacteria and preventing STIs^40,41^. The difference of nanh3 loads found in HPV16 infection is intriguing, are needed new approaches involving vaginal microbiota and HPV16, since this genotype is responsible for the most cases of high-grade intraepithelial lesions and invasive cervical carcinomas^42^.

Human papillomaviruses modulate the host immune response by blocking immune-related gene expression and immune signaling pathways and different genotypes may act by different pathways^43^. In addition, cervical microbiome and cytokine profile showed notably different in all stages of the natural history of cervical cancer^44^. Thus, bacterial species may interact with immune signaling during HPV infection contributing to persistence and progression^44^. On the other hand, a consequence of HPV immune evasion is the imbalance in the vaginal microbiota due the reduced amino acid source sustaining the survival of *Lactobacillus* species^45^. Therefore, the association between BV and HPV seems to be bi-directional. In fact, a study showed that the stability of vaginal microbiome is necessary to maintenance of immune surveillance for HPV16 clearance^46^. In this sense, the differences in the load of nanH3 gene in the presence of HPV16 lead to hypothesize that the vaginal microenvironment would have a different modulation in the presence of different genotypes and the changes in the immune response might create a more permissive microenvironment for bacterial overgrowth.

*Gardnerella* spp. is present in virtually all cases of BV, the most frequent dysbiosis of the vaginal microbiota^47^. In this study, an association between the nanH3 gene and BV was observed. Robinson et al. suggested the potential use of nanH3 as a molecular diagnostic marker of BV, such PCR test showing 80.95% sensitivity and 78.26% specificity compared with the Nugent score for BV diagnosis^25^. Although the association, in this study values of sensitivity and specificity were lower than the mentioned study. It is worth mentioning that studies differ in terms of prevalence of BV and number of study participants.

Besides the association between nanH3 gene and BV, the load of the gene was significantly increased in BV compared to normal microbiota. As previously discussed, sialidases have deleterious effects in the vaginal microenvironment^20–22,35–37^. Recently, our study group showed that sialidase activity in molecular-BV, assessed by V3–V4 16S rRNA sequencing, is associated with changes in bacterial components of the local microbiome as well as increased sialidase levels^48^. Indeed, sialidase activity in cervicovaginal fluid already was demonstrated in the presence of microscopy-detected BV^49,50^. In fact, sialidase activity could promote the growth or colonization of *Prevotella*, *Bacteroides* as well as *G. vaginalis*, vaginal bacterial sialidase producers^51,52^, being the production of sialidase an important step in the biofilm formation^24^. Such mechanism reinforces the role of *Gardnerella* spp. as the scaffold on vaginal mucosa for attachment of other bacterial species, which can start to form biofilm^53^, a condition already associated with refractory treatment of BV and persistence of BV-associated bacteria after treatment^23,54^.

Although the amount of sialidase-encoding nanH3 gene did not differ between clearance and persistence in the presence of all hrHPV infection, the loads are high in the persistent group and there is a difference among the groups when considering only HPV16 infection. A limitation of this analysis is due the low sample size when stratifying each genotype. Furthermore, such results contribute to better understanding the role of *G. vaginalis* sialidase-encoding gene in BV, since this condition seems have an important role in the persistence and progression of HPV infection. Thus, microbial products are promising tools for identifying women with increased risk for HPV persistence.

## Methods

### Data collection, sampling and laboratorial procedures

Between 2012 to 2014, 1635 reproductive-aged HIV-negative women screened for HPV infection in Botucatu, SP, Brazil. A sociodemographic, behavioral, gynecological history and clinical data were obtained during a face-to-face interview using a structured questionnaire and were entered into a Microsoft Excel spreadsheet (Microsoft Corporation, Redmond, WA, USA). HPV positive women were then invited to a follow-up within 12 months. All participants were informed about the procedures of study and informed consent was obtained from all subjects as all reached the age of majority (18 years-old). All methods were carried out in accordance with relevant guidelines and regulations and this current study was reviewed and approved by the Ethics Committee of Botucatu Medical School (Approval number: 5.660.472) and the samples included in the present study belongs to the biorepository of principal study (Biorepository approved number: 3.140.843).

Same sampling procedures during physical examination were conducted by nurses/physicians at enrolment and at follow-up visit. After speculum insertion, vaginal content was obtained with swabs for smearing into microscopic glass slides for Nugent scoring, after gram staining^15^. Mid vaginal wall was utilized for *Trichomonas vaginalis* culture in Diamond’s medium^55^. Samples obtained with endocervical brushes were stored at −20 ºC until *Chlamydia trachomatis* and *Neisseria gonorrhoeae* detection, as described previously^55,56^ and for HPV detection and genotyping using the Linear Array HPV Genotyping kit (Roche Molecular Systems, Pleasanton, CA, USA). For cervicovaginal fluid sampling, 3 ml of sterile 0.9% NaCl was inserted into vaginal wall, homogenized with cervicovaginal secretion and recovered using a plastic sterile pipette. The cervicovaginal samples were stored at −20 ºC until genomic DNA extraction and *G. vaginalis* nanH3 gene absolute quantification.

Genomic DNA extraction of cervicovaginal fluid was performed using the UltraClean Soil DNA Isolation kit (MoBio Laboratories, Carlsbad, CA, USA) as recommended by the manufacturer. Extracted DNA was quantified using an Epoch spectrophotometer (Biotek, Winooski, VT, USA) and the quality of the extraction was confirmed by the 260/280 nm absorbance ratio.

A cloning step was performed to obtain the plasmid DNA with *Gardnerella* nanH3 gene, through polymerase chain reaction (PCR) products from clinical samples using the primers *Gardnerella* nanH3 F (5′-CAGTTCCAATGGAAGTGTGC-3′) and *Gardnerella* nanH3 R (5′-AGCATCTGGGAATGCTCTTG-3′) with 51 ºC of annealing temperature. Expected amplicon size was 322 bp for nanH3 gene, confirmed in 1.5% agaroses gel band^25^. Positive samples had the amplicon purified using Illustra GFX PCR DNA and Gel Band Purification Kit (GE Healthcare UK Buckinghamshire, UK) and sequenced by Sanger method in ABI 3500 (Applied Biosystems, Foster City, CA, USA) and confirmed by blast (https://blast.ncbi.nlm.nih.gov/Blast.cgi#alnHdr_2235500528). The amplified products were used for the ligation step using the CloneJET PCR Cloning Kit (Thermo Fisher Scientific, Carlsbad, CA, USA), cloned into *Escherichia coli* DH5-α (Invitrogen, Carlsbad, CA, USA). Plasmid DNA was extracted using GeneJET Plasmid Miniprep Kit (Thermo Fisher Scientific, Carlsbad, CA, USA).

The absolute quantification of nanH3 gene was performed by quantitative real-time PCR (qPCR) using 2× qPCRBIO SyGreen Blue Mix Hi-ROX (PCR Biosystems, London, UK), primers *Gardnerella* nanH3 F and *Gardnerella* nanH3 R under the following cycling conditions: 95 °C for 2 min, 25 cycles of denaturation at 95 °C for 5 s, annealing at 60 °C for 25 s, and an extension at 95 °C for 15 s followed by a dissociation stage: 95 °C for 15 s; 60 °C for 1 min and 95 °C for 15 min, according to the manufacturer's recommendations, performed in a StepOnePlus Real-Time System (Thermo Fisher Scientific, Waltham, MA, USA). Samples with a melting temperature value of 81 °C + / − 0.5 °C were considered positive for nanH3 gene. For constructing the standard curve, three plasmid dilutions (3.05E+05 copies/µL, 3.05E+07 copies/µL and 3.05E+09 copies/µL) were utilized for sample cycle threshold interpolation. Loads of nanH3 gene were expressed as the number of copies per volume (µL) of cervicovaginal fluid.

### Selection of participants and group assignments

In the first visit, 544 (33.27%) women were positive for any HPV infection, of which 413 (25.26%) tested positive for hrHPV genotypes and were recruited for the follow-up visit after 12 months. Four hundred sixty-two (27.65%) women returned to the follow-up. For the present study, women positive for *Chlamydia trachomatis* (138, 8.44%), *Trichomonas vaginalis* (23, 1.41%), *Neisseria gonorrhoeae* (2, 0.12%) and only low-risk HPV (131, 8.01%) were not included in the analysis. Five women were excluded of this study due conflicting data in the spreadsheet database. From that, 245 (14.96%) women were hrHPV positive (HPV16, HPV18, HPV31, HPV33, HPV35, HPV39, HPV45, HPV51, HPV52, HPV56, HPV58, HPV59, HPV68, HPV73, HPV82), however, 12 samples were excluded due insufficient volume of cervicovaginal fluid for laboratorial analysis of the current study. Therefore, 233 cervicovaginal fluid samples were included for this study analysis. Participants were assigned at two groups according to the status of hrHPV infection at enrolment and at follow-up: ‘clearance group’ comprised participants who tested negative for any hrHPV at follow-up and in ‘persistence group’ were assigned participants positive for hrHPVs genotype at first visit and follow-up, matched with those detected at follow-up. For clearance group, 21 women who showed clearance of baseline genotype and had a new genotype detected at follow-up were not included.

### Statistical analysis

For sociodemographic, behavioral and clinical variables comparisons between the clearance and persistence groups, chi-squared or Fisher’s exact were utilized for categorical variables and Mann–Whitney tests was utilized for continuous variables. The loads and presence of nanH3 gene were compared between the study groups using, respectively, Mann–Whitney and chi-squared tests or Fisher’s exact test. All analyses considered a P-value < 0.05 to be statistically significant and were performed using GraphPad Prism software (version 6.0, GraphPad, CA, USA).

### Supplementary Information

Supplementary Table S1.

## Data Availability

The datasets used and/or analyzed during the current study are available from the corresponding author on reasonable request.
