# Supplementary material for: Cervicovaginal Gardnerella sialidase-encoding gene in persistent human papillomavirus infection
Source: Sci Rep. 2023 Aug 31;13:14266. doi: 10.1038/s41598-023-41469-8 (PMC10471596; doi:10.1038/s41598-023-41469-8)
Supplement: Supplementary file 1 — Supplementary Table S1. [file 41598_2023_41469_MOESM1_ESM.docx]

**Table Suppl 1: Frequency of each hrHPV detected as single or mixed infection**

| **Baseline hrHPV genotypes detected** | **Frequency in single infection (n=164)** | **Frequency in mixed infection (n=48)** | **Total of infection for each hrHPV (212)** |
| --- | --- | --- | --- |
| HPV16 | 43 (26.22) | 21 (43.75) | 64 (30.19) |
| HPV31 | 15 (9.15) | 13 (27.08) | 28 (13.21) |
| HPV52 | 15 (9.15) | 11 (22.92) | 26 (12.86) |
| HPV51 | 17 (10.37) | 5 (10.42) | 22 (10.38) |
| HPV58 | 11 (6.71) | 11 (22.92) | 22 (10.38) |
| HPV45 | 14 (8.54) | 7 (14.58) | 21 (9.91) |
| HPV18 | 12 (7.32) | 8 (16.67) | 20 (9.43) |
| HPV56 | 8 (4.88) | 8 (16.67) | 16 (7.55) |
| HPV35 | 6 (3.66) | 7 (14.58) | 13 (6.13) |
| HPV59 | 6 (3.66) | 7 (14.58) | 13 (6.13) |
| HPV73 | 7 (4.27) | 5 (10.42) | 12 (5.66) |
| HPV68 | 5 (3.05) | 6 (12.50) | 11 (5.19) |
| HPV82 | 3 (1.83) | 6 (12.50) | 9 (4.25) |
| HPV39 | 1 (0.61) | 6 (12.50) | 7 (3.30) |
| HPV33 | 1 (0.61) | 2 (4.17) | 3 (1.42) |
